# Challenging claimed benefits of soil carbon sequestration for mitigating climate change and increasing crop yields: Heresy or sober realism?

**DOI:** 10.1111/gcb.16640

**Published:** 2023-02-26

**Authors:** David S. Powlson, Marcelo V. Galdos

**Affiliations:** ^1^ Sustainable Soils & Crops Rothamsted Research Harpenden UK

## Abstract

This is a commentary on the paper in GCB by Moinet et al. (2023) entitled “Carbon for soils, not soils for carbon”. The paper challenges two claims often made for soil carbon sequestration: (1) Sequestration of C in agricultural soils can make a substantial contribution to climate change mitigation. (2) Increasing SOC will routinely lead to increased crop yields and contribute to global food security.

There is overwhelming evidence that increasing the organic carbon (C) content of cropland soil that has become depleted in C improves its physical, chemical and biological properties, with benefits for the growth of crop roots and the functioning of soils in the wider environment (King et al., 2020; Kopittke et al., 2022; Lal 2020). This is entirely uncontroversial. It is currently relevant because there is evidence that soil organic carbon (SOC) in many cropland soils globally is declining (Sanderman et al., 2017) and is vulnerable to further loss from climate change (Lugato et al., 2021). It may, therefore, seem counterintuitive, and even heretical or downright unhelpful, for a paper to challenge two widely stated claims connected with SOC as is done in the paper entitled “Carbon for soils, not soils for carbon” by Moinet et al. (2023). The two claims challenged by the authors are:

1. Sequestration of C in agricultural soils can make a substantial contribution to climate change mitigation.
2. Increasing SOC will routinely lead to increased crop yields and contribute to global food security.

The authors are particularly critical of these two assertions being combined to make the claim that SOC sequestration is a “win‐win” strategy. They point out that climate change and food security have both been described as “wicked problems” of “daunting complexity” so blanket solutions that claim to solve both “should prompt some degree of scepticism.” In this commentary, we draw attention to the conclusions of Moinet et al. (2023) and add some suggestions of our own.

## 1 LIMITATIONS OF SOC SEQUESTRATION FOR CLIMATE CHANGE MITIGATION

Moinet et al. (2023) cite numerous publications and international initiatives that give prominence to SOC sequestration as a highly significant contributor to climate change mitigation. In contrast to the enthusiasm for this approach, they review some of the misconceptions regarding the role of soil C sequestration. They point out the many sources of uncertainty in attempting to estimate the quantity of additional C that could be transferred from atmospheric CO_2_ to stabilized SOC through alterations in land management such as no‐till, cover crops, improved grazing management or agroforestry. They focus particularly on the issue of SOC saturation. Evidence from all long‐term studies shows that, with the exception of peat formation, SOC does not increase indefinitely but tends towards a new quasi‐equilibrium value with the annual accumulation rate slowing over time (e.g. Poulton et al., 2018). For convenience, when comparing rates of SOC increase between management practices, it is common to express the rate on an annual basis (i.e. as kg C per ha per year to a specified soil depth). But failing to recognise the effect of saturation, and the resulting slowing of SOC accumulation, can lead to serious over‐estimation of C sequestration potential.

To meet the Paris Agreement aim of limiting the mean global temperature increase by 2100 to 1.5°C requires a cumulative decrease in CO_2_ emissions by then of around 3000 Gt CO_2_. Moinet et al. (2023) consider a range of assumptions in published data regarding SOC sequestration rates in agricultural soils. Taking one example, the assumption of no saturation and a constant sequestration rate over time gives an estimated C sequestration by the year 2100 of 257 Gt CO_2_. However, introducing two different but reasonable assumptions about the slowing of C sequestration as SOC content increases reduces this to 121 or 49 Gt CO_2_. Thus, ignoring saturation leads to an overestimation of C sequestration of 53%–81%. They conclude that taking account of saturation and using more realistic assumptions than is often done, the contribution from SOC sequestration by 2100 is likely to be in the range of 4% to less than 1% of global emissions. While any contribution is welcome, this analysis is valuable in putting it into a quantitative perspective and is in line with other recent estimations approached from different viewpoints, for example Janzen et al. (2022).

## 2 SOC AND FOOD SECURITY

Does increasing SOC lead to increased crop yields? This is a notoriously difficult question to answer. In virtually all long‐term agricultural experiments globally, treatments giving higher crop yields (e.g. from N fertilizer application) have slightly higher SOC than in lower yielding treatments (Ladha et al., 2011; Tang et al., 2022). But what is cause and what is effect? Higher yielding crops deposit slightly more organic C into the soil in roots, root exudates and above‐ground residues leading to increased SOC in the long term (Jenkinson et al., 1992; Tang et al., 2022). So SOC and yield tend to be correlated but, as is well known, correlation does not equal causation.

Moinet et al. (2023) collate data from 21 meta‐analyses relating to SOC and crop yields. Two main approaches have been used to seek causality, both of which have serious drawbacks because any direct effect of SOC is confounded with other factors:

1. Comparing crop yields at a range of sites that differ in SOC content (space‐for‐time comparisons).
2. Comparing crop yields at single sites where SOC has been increased by addition of manure or other organic inputs.

In the first approach sites that differ in SOC content often also differ in other respects, especially soil type. Soils with a higher clay content generally have higher SOC so any effect on crop yield could either be from SOC or from a factor associated with clay content such as increased availability of water or nutrients. In the second approach crop growth and yield is likely to be influenced by release of nutrients from the manure in addition to any effect directly related to soil C. The studies reviewed showed examples of crop yields being increased, decreased or unchanged where SOC was greater. In cases where additional nutrient supply from mineralization of manure or soil organic matter was taken into account there were no examples of an overall positive effect of SOC on yield. It was therefore concluded that this aspect of the “win‐win” assertion was not substantiated.

Moinet et al. (2023) point out some nuances within the overall data and these deserve rather more emphasis. First, in sandy soils, there was a greater tendency for higher yields to be associated with increased SOC. Second, crops with a short growing season were more likely to show increased yield where SOC was higher. This was previously noted by Hijbeek et al. (2017) in an analysis of crop yields in some European long‐term experiments. It is also seen in the comparison of autumn‐sown wheat and spring‐sown barley in the long‐term experiments at Rothamsted, UK (Macdonald et al., 2017). This finding may be particularly relevant for tropical situations where it is common to grow two or more crops per year instead of one that is the norm under temperate conditions. So, although the “win‐win” from increased SOC may not be correct overall, in some situations of global significance for food security the yield “win” is valid.

In many agricultural situations globally, organic resources such as manure or crop residues are in short supply and have competing uses, so decisions have to be made on how best to use them. A focus on maximising C storage for climate change mitigation would lead to prioritising their use on clay soils as these offer greater SOC sequestration capacity than sandy soils. However, the meta‐analyses provide evidence that greater yield benefits are likely in sandy soils. Hence situation‐specific assessments are needed rather than a blanket assumption of “win‐win”.

## 3 WHAT THE PAPER DOES *NOT* SAY

Papers that challenge orthodox opinions can easily be misunderstood or misinterpreted and it is likely that this will be the case with Moinet et al. (2023). It is important to point out that the authors do *not* say that SOC is unimportant—quite the opposite. Their key points are:

1. The extent of climate change mitigation attainable by sequestering additional organic C in agricultural soils is very limited. But of course, any such benefit is welcome.
2. Evidence does not support the idea that increased SOC inevitably leads to increased crop yields—it does in some situations but by no means all.

Regarding crop production and food security, we would emphasise two additional points. First, where SOC has been increased, the soil is likely to supply more nutrients, especially N. This means that less inorganic N fertilizer is required to attain the desired yield thus saving greenhouse gas emissions associated with N fertilizer manufacture and so contributing indirectly to climate change mitigation. Second, in most agricultural situations increased SOC almost certainly contributes to the sustainability of crop production mainly through maintaining soil physical conditions that are suitable for root growth and water infiltration and retention (King et al., 2020). This is difficult to prove unequivocally but is a strong reason to adopt management practices that maintain or increase SOC, even where there is no short‐term yield benefit.

Our recommendation regarding SOC management is in line with the conclusions of Moinet et al. (2023). It is more helpful to focus on the role of SOC in climate change *adaptation* and the *resilience* of agricultural systems to adverse weather conditions (Droste et al., 2020) rather than continuing to concentrate narrowly on mitigation through C sequestration. This will contribute to agricultural and environmental sustainability. Hence “Carbon for soils, not soils for carbon”.

## Data Availability

Data sharing not applicable to this article as no datasets were generated or analysed for this commentary.
